# Graves' Disease and Acute Rheumatism

**Published:** 1908-02-29

**Authors:** 


### Clinical Points.

### GRAVES' DISEASE AND ACUTE RHEUMATISM.

It seems very unlikely that acute rheumatic fever is in any way causally related to exophthalmic goître; for if this were so, the latter disease should be very much more common in London than it is. Nevertheless, in the analysis of Graves' disease cases, it is surprising how often there is a history of acute rheumatism, at least in the same family if not actually in the same person. About one-third of all the exophthalmic goître cases have had either acute articular rheumatism itself, or one of the other manifestations of the same disease—erythema nodosum, for example, or chorea.

This is a point upon which there is yet little information in the text-books, although it has been clinical knowledge for years. The French have been writing a good deal about it lately, as though it were a new thing; but there were papers upon the subject long ago in England, and it is a pity that they are not more widely known.

If the exophthalmic goître patient has not herself suffered from rheumatic fever, there is often a strong family history of the latter. Occasionally two sisters are in the wards of a large hospital at the same time, the one suffering from acute rheumatism, the other from Graves' disease; and there can be no doubt of the tendency of the two complaints to be associated with one another, even if they are not directly related to each other.

There is a tendency to scoff at the word "diathesis," but it is undoubtedly useful; and, indefinite though it may be, it shortly summarises what one means when one says that a person is particularly prone to suffer from some special kind of disease. There is not the least doubt that some persons and families are much more liable than others to suffer from rheumatic fever or its equivalents; in other words, certain people have the "rheumatic diathesis." In the same way, but to a much less obvious extent, certain families seem to have the "Graves' disease diathesis." It seems certain, too, that exophthalmic goître cases are specially prone to have the "rheumatic diathesis." This is probably the basis of the association of the two diseases in the same patient. There is little reason to suppose that acute rheumatism and Graves's disease have a common cause; but there is much reason to believe that a person who is liable to develop exophthalmic goître is also particularly liable to acute rheumatic affections.

It is impossible to give full illustrations of this, and even to analyse the statistics upon the subject would be out of place here. The following case, however, is a good example of the association in question, in that exophthalmic goître developed actually during convalescence from acute rheumatism. The closeness of the association here is greater than in most instances; more often a person who has expophthalmic goître either has had rheumatic fever in former years, or develops it when the Graves' disease has already been in existence for some time.

The patient was a man aged 44. He had been quite well, and a soldier, up to 29, when he had his first attack of typical rheumatic fever, for which he was laid up for many weeks. Almost every year from that time he had had a slight return of rheumatic symptoms, and he was now admitted for an attack as severe as the one he had when he was 29. The joint pains were extremely acute, but they were greatly lessened by full doses of sodium salicylate. A fortnight after the joint pains were thus relieved he began to complain of nervousness, and of great agitation from slight causes. His heart was also a source of trouble to him, palpitating violently upon occasions without apparent reason. The eyes, a few days later, assumed the typical stare of Graves' disease; there was actual as well as apparent exophthalmos. Stellwag's sign was well marked; von Graefe's was absent. Attention was thus called to the thyroid gland, and this was found to be uniformly enlaged, soft, and elastic. How long it had been so it was not possible to determine; presumably the enlargement was recent. There was the usual tremor of the hands. The heart was beating at from 140 to 150 times per minute. In short, the patient had developed for the first time typical exophthalmic goître whilst under observation for acute rheumatic fever.

It is to be hoped the effects of the conjunction of the two complaints in this patient are seldom seen in others. His condition went from bad to worse. He lived for six weeks longer, but with one grave symptom supervening upon another. Œdema of the feet set in first; then ascites; then severe diarrhœa with pyrexia—a pair of symptoms which are always of the gravest import in exophthalmic goître cases; the heart rapidly dilated and jaundice developed; and for some time before death there was pericarditis. At the post-morten examination there were the combined lesions of Graves' disease and of rheumatic fever. To the former were due the enlargement of the thyroid and thymus glands, the swollen lymphoid follicles of the intestine, the jaundice, and part of the dilatation of the heart. To the rheumatism were due the acute pericarditis with effusion, the granulations on the aortic and mitral valves, the myocarditis and part of the cardiac dilatation, the œdema of the legs, the nutmeg liver and the ascites.

It is well known that Graves' disease may cause some cardiac hypertrophy, and that it may be the source of blowing systolic bruits in every region of the heart. It may, therefore, be very difficult in some cases, where there has also been rheumatic fever, to determine precisely how much of cardiac anomaly may be due to the Graves's disease, and how much to rheumatic lesions of the myocardium and valves.

The case which has been quoted is certainly an extreme one, but the short account we have given of it will have served its purpose if it assists in calling renewed attention to the liability of exophthalmic goître patients to suffer from acute rheumatism and its effects.

